# Pathogen distribution in pulmonary infection in chinese patients with lung cancer: a systematic review and meta-analysis

**DOI:** 10.1186/s12890-023-02681-4

**Published:** 2023-10-23

**Authors:** Yanyan Wang, Jia Li, Qinqin Wu, Qin Chang, Shuming Guo

**Affiliations:** Department of Respiratory and Critical Care Medicine, Linfen Central Hospital, 041000 Linfen, China

**Keywords:** Lung cancer, Meta-analysis, Pathogen distribution, Pulmonary infection, Gram-negative bacteria, Gram-positive bacteria, Fungi

## Abstract

**Background:**

The immunity of patients with lung cancer decreases after treatment; thus, they are easily infected with pathogenic bacteria that causes pulmonary infections. Understanding the distribution characteristics of pathogenic bacteria in pulmonary infection in patients with lung cancer after treatment can provide a basis to effectively prevent infection and rationally use antibacterial drugs. However, no meta-analyses have assessed the distribution characteristics of pathogenic bacteria in mainland China. Therefore, our meta-analysis aimed to investigate the pathogen distribution in pulmonary infection in Chinese patients with lung cancer.

**Methods:**

A literature search was conducted to study the pathogen distribution in pulmonary infection in Chinese patients with lung cancer between January 1, 2020 and December 31, 2022, using English and Chinese databases. The relevant data were extracted. The meta-analysis was performed using a random-effects model ( I^2^ > 50%) with 95% confidence intervals for forest plots. Data were processed using RevMan 5.3.

**Results:**

Fifteen studies (2,683 strains in 2,129 patients with pulmonary infection were cultured) met the evaluation criteria. The results showed that Gram-negative bacteria had the highest detection rate (63%), followed by Gram-positive bacteria (23%), and fungi (12%). Among the Gram-negative bacteria detected, the distribution of the main pathogenic bacteria was *Klebsiella pneumonia* (17%), *Pseudomonas aeruginosa* (14%), *Escherichia coli* (13%), *Acinetobacter baumannii* (7%), *Enterobacter cloacae* (4%), and *Hemophilus influenza* (4%). Moreover, the prevalence of pulmonary infections after chemotherapy (53%) was significantly higher than that after surgery (10%), P < 0.05.

**Conclusions:**

The prevalence of pulmonary infections after treatment, especially after chemotherapy, is high in Chinese patients with lung cancer, and Gram-negative bacteria are the predominant pathogens. Further studies are needed to monitor the prevalence of pulmonary infections and pathogen distribution in lung cancer patients in mainland China.

## Background

Lung cancer is a malignant tumor of the respiratory system [1]. However, it is often neglected because of its insidious and non-specific symptoms in the early stages [2]. Most patients are diagnosed at a late stage; therefore, treatment and prognosis are poor [3]. Lesions can be effectively removed by surgical treatment which can also prevent the progression of lung cancer [4]. However, owing to the extensive surgical trauma, the normal environment of the patient’s chest is easily destroyed, resulting in a large amount of postoperative gas and fluid accumulation [5]. Moreover, postoperative immunity is reduced and postoperative complications occur more frequently [6]. Moreover, Among these complications, pulmonary infection is the most common, and it can affect the prognosis and outcomes of the patients.

Chemotherapy is the classic treatment for early stage lung cancer, which often has no symptoms, thus missed during surgeries [7]. Recently, immunotherapy has become a major treatment option for lung cancer [8–10]. However, chemotherapy can also cause adverse effects and complications, such as liver and kidney damage and gastrointestinal symptoms [11]. Furthermore, it can reduce patient immunity to varying degrees, thus inducing pathogen infections [12], which not only affect the quality of life of patients, but also increase their economic burden. Surgery is also used as a treatment for lung cancer. However, the operation is invasive, causing great trauma to patients, and the postoperative sputum excretion ability of patients is poor, and respiratory secretions cannot be discharged in time, which is easy to increase the risk of pathogen infection [13].

Patients with lung cancer have very low immunity, especially after chemotherapy or surgery; thus, they are more likely to develop pulmonary infection after treatment [13, 14], which is a common cause of death [15]. Therefore, it is necessary to monitor the prevalence of lung cancer complicated by pulmonary infections and the distribution of pathogenic bacteria after treatment. Currently, there are reports of Chinese patients with lung cancer complicated by pulmonary infections. However, systematic reviews and meta-analyses are rare. Some reports in China have evaluated the types of pathogenic bacteria in lung cancer patients [16–30]. *Pseudomonas aeruginosa*, *Streptococcus pneumoniae*, *Klebsiella pneumonia* and other pathogenic bacteria are relatively common. However, there are regional differences or different treatment methods in these studies, so the reported pathogens do not fully demonstrate the distribution of pathogenic bacteria in China.

Therefore, our systematic review and meta-analysis summarized the previously published data of pulmonary infection in Chinese patients with lung cancer to improve the current understanding of its pathogen distribution. This study could help in developing effective prevention strategies and treatment guidelines to promote disease management.

## Materials and methods

### Literature search strategy

A systematic review was conducted according to the methods and recommendations of the PRISMA (Preferred Reporting Items for Systematic Reviews and Meta-Analyses) extension statement. We searched English language databases (PubMed, Google Scholar, Cochrane Library, and Clinical Trials) and Chinese language databases (CNKI, Cqvip, WANFANG data, and Baidu scholar) to retrieve articles about the pathogen distribution of pulmonary infection in lung cancer patients that had been published between January 1, 2020, and December 31, 2022. The search method adopted a combination of subject words and free words, and the search terms included “lung cancer,” “infection,” “etiology,” “nosocomial infection,” “pathogen,” “pathogen distribution,” “China,” and “Chinese,” or variants and combinations of these keywords. The reference lists of the included studies were reviewed to identify other potentially eligible studies.

### Inclusion and exclusion criteria for articles

Fifteen selected publications that met established inclusion criteria were independently evaluated and data extracted by two reviewers. They were evaluated and data extracted based on the information provided in the title and/or abstract as well as their full text, if available in English. Furthermore, these publications contained data on nosocomial infections in lung cancer patients after chemotherapy or surgery in mainland China regardless of sex and age. Moreover, the patients in these studies were diagnosed using clear diagnostic criteria for nosocomial infections. The following studies were excluded from our systematic review and meta-analysis: (1) non-research-based publications, such as reviews, press releases, newsletters, and forums; (2) republished studies; (3) studies with unclear diagnostic criteria or unconventional diagnostic tools; (4) studies on treatment of uncertainty measures; (5) non-detailed analysis of the type of pathogen infection in patients; and (6) publications lacking precise case counts, making it impossible to calculate the corresponding indices.

### Data extraction and quality assessment

Corresponding data were extracted from studies by two reviewers that met the inclusion criteria and included the following information: first author, year of publication, number of patients, number of strains, therapeutic measures (chemotherapy or surgery), population pathogens (Gram-negative bacteria, Gram-positive bacteria, or fungi), pathogenic bacteria, and prevalence (population pathogen, therapeutic measures, and pathogenic bacteria). The quality of the selected publications was estimated according to the criteria of the Grading of Recommendations Assessment, Development, and Evaluation method. Studies were assigned 0–4 points based on the scoring criteria. Studies with 3–4 points were considered of high quality, those with 2 points were deemed to be of moderate quality, and those with 0–1 points were considered to be of low quality. Moreover, studies with scores of 0 were excluded. The included studies were assessed in a single-blinded manner.

### Statistical analysis

A random-effects model was used for the eligible studies. The meta-analysis was performed using a Review Manager 5.3 (Copenhagen: The Nordic Cochrane Centre, The Cochrane Collaboration, 2014). Forest plots were used to summarize the estimates with 95% confidence intervals (CIs). The heterogeneity index among the included studies was determined using a Cochran’s Q test (chi-square test) and Higgins’ I^2^ statistics, which if significant (I^2^ > 50%), a random effects model was used. Potential publication bias was assessed using funnel plots. The statistical significance was set at P < 0.05, and the 95% CIs were reported.

## Results

### Description of studies

**Fig. 1:**
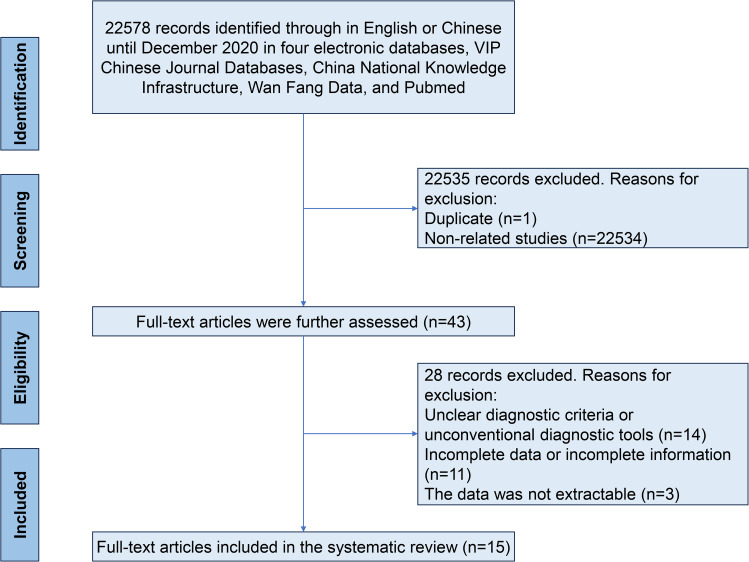
A flow chart for screening eligible studies

Based on the database search strategies, 22,578 Chinese and English articles were identified. Subsequently, 43 full-text articles were selected, and review papers, duplicate citations, and studies irrelevant to the current meta-analysis were removed. After excluding 28 articles with incomplete data, 15 met the inclusion criteria and were included in this systematic review (Fig. 1). All included studies were sampled between 2015 and 2022. In total, 2,683 strains detected in 2,129 patients with pulmonary infection were cultured. According to our quality criteria, 0 publications were of high quality (4–5 points), 13 were of moderate quality (2–3 points), and 2 were of low quality (1 point). A cross-sectional review of all articles was performed (Table 1).

**Table 1:** Included publications from mainland China

Reference	Publication Year	No. examined	No. positive	No. Strains	No. gram-negative bacteria Strains	No. gram-positive bacteria Strains	No. fungi Strains	Therapeutic measures	Study design	Score
Aixian Hu [16]	2022	730	82	136	71	55	10	surgery	Cross sectional	3
Biao Xu [17]	2021		246	309	189	91	29	surgery	Cross sectional	2
Fei Gao [18]	2022	358	32	49	31	15	3	surgery	Cross sectional	2
Ge Meng [19]	2022	114	34	49	34	13	2	chemotherapy	Cross sectional	3
Hongxiao Tuo [20]	2022		100	108	66	32	10	surgery	Cross sectional	2
Hongyan Xu [21]	2022		349	412	247	124	41	surgery	Cross sectional	1
Jianying Wang [22]	2022		170	202	127	56	19	chemotherapy	Cross sectional	2
Jingxiang Yang [23]	2020		120	206	158	16	32	chemotherapy	Cross sectional	3
Jun Zhu [24]	2017		324	396	280	60	56	chemotherapy	Cross sectional	3
Miao Zhang [25]	2015		65	103	79	8	16	chemotherapy	Cross sectional	1
Qiuhong Bao [26]	2019	411	184	204	84	32	36	chemotherapy	Cross sectional	3
Yanli Zhang [27]	2020		136	151	106	26	19	chemotherapy	Cross sectional	3
Zewen Zhu [28]	2021		159	202	114	56	32	chemotherapy	Cross sectional	2
Zhen Wang [29]	2021	250	25	37	27	9	1	surgery	Cross sectional	3
Zhihui Liu 30]	2021	124	103	119	65	19	35	chemotherapy	Cross sectional	2

### Type of pathogenic infections

**Fig. 2:**
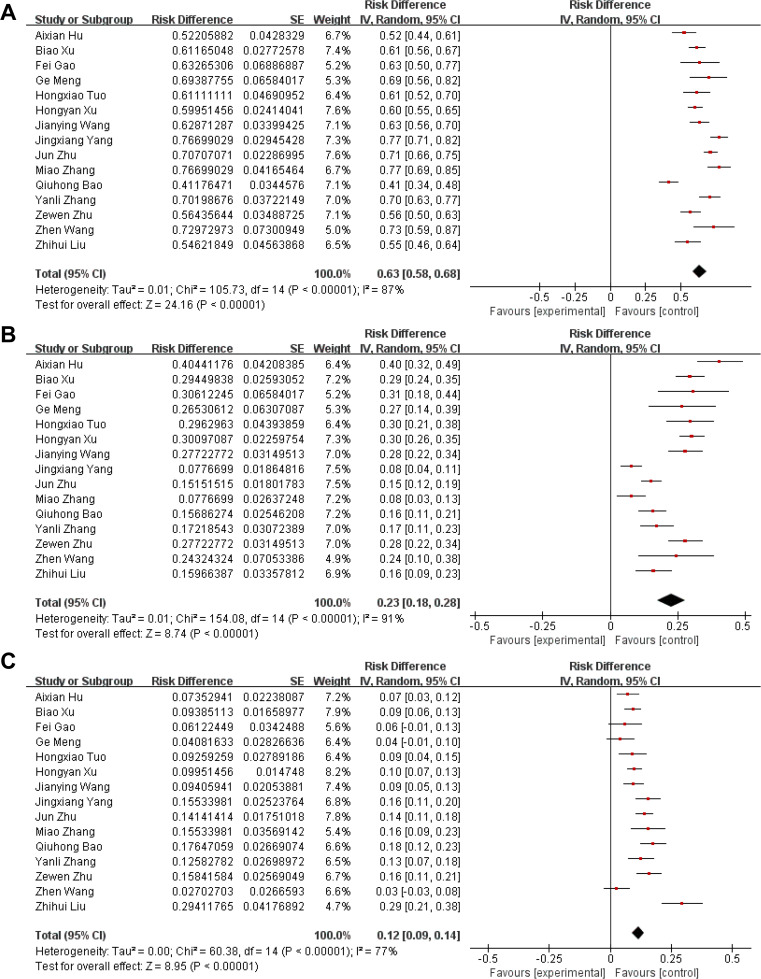
A forest plot of the distribution characteristics of pathogenic bacteria in patients with pulmonary infection. **(A)**: Gram-negative bacteria. **(B)**: Gram-positive bacteria. **(C)**: Fungi

Among the 2,683 cultured strains, the pooled detection rate of Gram-negative bacteria was 63% (95% CI: 58–68; χ^2^ = 105.73; I^2^ = 87%; P < 0.00001) (Fig. 2A), and the distribution of the main pathogenic bacteria was *Klebsiella pneumonia* (17%), *Pseudomonas aeruginosa* (14%), *Escherichia coli* (13%), *Acinetobacter baumannii* (7%), *Enterobacter cloacae* (4%), and *Hemophilus influenza* (4%) (Table 2). The detection rate of Gram-positive bacteria was 23% (95% CI: 18–28; χ^2^ = 154.08; I^2^ = 91%; P < 0.00001) (Fig. 2B), mainly *Staphylococcus aureus* (9%), *Staphylococcus hemolyticus* (4%), Streptococcus (4%), and *Staphylococcus epidermidis* (1%) (Table 2). The detection rate of fungi was 12% (95% CI: 9–14; χ^2^ = 60.38; I^2^ = 77%; P < 0.00001) (Fig. 2C), mainly *Candida albicans* (6%), *Candida tropicalis* (3%), and *Candida globus* (2%) (Table 2).

**Table 2:** Detection and distribution of pathogenic bacteria

Pathogenic bacteria		No. studies	No. positive	No. Strains	% (95%, CI)	Heterogeneity		
						Chi^2^	P-value	I^2^
Gram-negative bacteria	Klebsiella pneumoniae	15	428	2,683	17 (12, 21)	201.65	< 0.00001	93%
	Pseudomonas aeruginosa	15	382	2,683	14 (12, 16)	32.71	0.003	57%
	Escherichia coli	12	356	2,444	13 (8, 18)	249.42	< 0.00001	95%
	Acinetobacter baumannii	15	202	2,683	7 (6, 9)	41.31	0.0002	66%
	Enterobacter cloacae	8	69	1,508	4 (3, 5)	10.20	0.18	31%
	Haemophilus influenza	6	82	1,782	4 (3, 5)	4.96	0.55	0%
Gram-positive bacteria	Staphylococcus aureus	15	269	2,683	9 (7, 12)	56.00	< 0.00001	75%
	Staphylococcus haemolyticus	11	104	1,832	4 (2, 6)	65.59	< 0.00001	85%
	Streptococcus	11	93	2,123	4 (3, 5)	14.01	0.17	29%
	Staphylococcus epidermidis	8	57	1,457	1 (0, 3)	2.77	0.91	0%
Fungi	Candida albicans	10	138	1,973	6 (4, 9)	77.60	< 0.00001	88%
	Candida tropicalis	8	60	1,885	3 (2, 4)	6.56	0.48	0%
	Candida globus	7	41	1,694	2 (1, 3)	12.12	0.06	50%

### Prevalence of different treatments

**Fig. 3:**
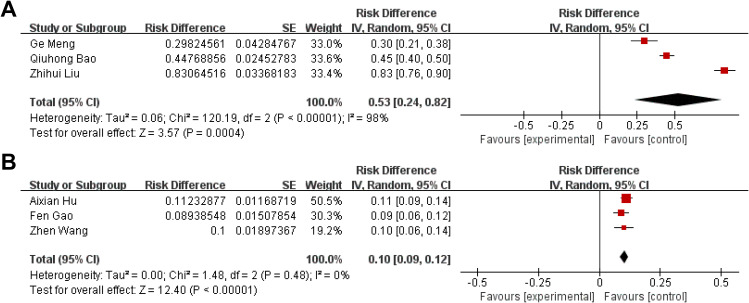
A forest plot of the prevalence of pulmonary infection in patients treated with chemotherapy or surgery. **(A)**: Chemotherapy. **(B)**: Surgery

The published articles were further divided into chemotherapy and surgery groups according to the treatment received. As shown in Fig. 3; Table 1, of the 15 included studies, six described the total number of patients and the number of patients who developed pulmonary infection after treatment. According to nine studies on 649 samples, the prevalence of patients treated with chemotherapy was 53% (95% CI: 24–82; χ^2^ = 120.19; I^2^ = 98%; P < 0.00001), and according to six studies on 1,338 clinical samples, the prevalence of patients who underwent surgery was 10% (95% CI: 9–12; χ^2^ = 1.48; I^2^ = 0%; P < 0.00001). The pooled prevalence between chemotherapy and surgery showed a statistically significant difference (P < 0.05).

## Discussion

Lung cancer is often neglected because of its insidious onset and absence of typical symptoms in the early stages [31]. Most patients are diagnosed at a late stage or even when a distant metastasis occurs, which seriously affects prognosis [32]. In China, surgery and chemotherapy are commonly used to treat lung cancer. However, these treatments affect the patients’ immunity; thus, they are easily infected with pathogenic bacteria that cause pulmonary infection, which is not conducive to recovery.

In this meta-analysis, the distribution of pathogenic bacteria in pulmonary infection in Chinese patients with lung cancer after treatment was analyzed, and the main pathogenic bacteria were Gram-negative bacteria, followed by Gram-positive bacteria and fungi. Granulocyte levels are reduced after surgery or chemotherapy, resulting in a decline in the patients’ immunity, which causes an infection [16, 19]. The results of the present study show that the detection rate of *Klebsiella pneumonia* was the highest, followed by that of *Pseudomonas aeruginosa*. These bacteria are normally present in the human intestinal and upper respiratory tracts [16, 21, 23]. Patients are more likely to become infected with these pathogenic agents if their resistance is compromised. In addition, in some hospitals in China, cancer patients are treated with broad-spectrum antibacterial drugs, and the inappropriate use of these drugs is likely to cause the imbalance of the patient’s body flora and increase the risk of drug resistance, and ultimately make patients more susceptible to infection with these pathogenic bacteria. Moreover, *Staphylococcus aureus* had the highest detection rate among the Gram-positive bacteria. Staphylococcus aureus, a normal human flora, can cause various infections when the immunity is compromised [19]. In addition, lung cancer lesions are prone to necrosis due to ischemia, cavity formation, can be secondary infection with these pathogens. And repeated hospitalization can also cause cross-pathogenic bacterial infection. Therefore, effective measures should be taken to reduce the risk of *Staphylococcus aureus* infections. The detection rate of fungal infections was 12%, which might have been related to the low immunity and the irrational use of antibiotics. Therefore, fungal infections should also be considered in the prevention of pulmonary infections.

We also analyzed the prevalence of pulmonary infections in patients treated with surgery or chemotherapy. The prevalence of chemotherapy was significantly higher than that of surgery. Chemotherapy inhibits their growth and reproduction, and kills the malignant ones [32]. Furthermore, it has curative effects on primary, subclinical, and metastatic lesions. However, while inhibiting the spread of cancer cells, chemotherapy has a myelo-suppressive effect, which decreases the total number of white blood cells and neutrophils, leading to an increased susceptibility to pulmonary infections [30]. Herein, the prevalence of pulmonary infection after chemotherapy was 53%. Therefore, we should pay attention to treating primary diseases, establishing supportive disinfection and sterilization measures, raising awareness of aseptic operations, and reducing pulmonary infection after chemotherapy. Moreover, the prevalence of pulmonary infections after surgery was 10%. In lung cancer patients, long-term gastric catheterization after surgery can damage the esophagus and gastric mucosa, which can easily cause the displacement of the gastrointestinal flora, thus increasing the probability of postoperative pulmonary infection [33]. Therefore, infection should be well prevented postoperatively; sputum culture and drug sensitivity tests should be performed in time for patients with suspected infection; and antibiotics should be applied reasonably to reduce the generation of drug-resistant strains.

This study had some limitations. First, Second, the data were limited, as only 15 publications met the inclusion criteria, including only three studies on the prevalence after surgery or chemotherapy. Second, all included studies were published in Chinese. Although these publications met the inclusion criteria, we hope that more English publications will be readily accessible later on. Third, the included studies could not effectively divide the geographical distribution, so this study lacks a comparison of infection among different administrative regions in China. And this study lacks a comparison of pathogenic bacteria among different ethnic groups in China. All these need to be further analyzed in order to better understand the distribution characteristics of pathogenic bacteria in Chinese lung cancer patients. Additional, when the infection rates of Gram-negative bacteria, Gram-positive bacteria and fungi were analyzed in this study, the results included in the study showed heterogeneity (I^2^ > 50%). This may be related to the fact that these studies came from different sample areas and populations, which will be further analyzed in future studies.

## Conclusion

The prevalence of pulmonary infections after treatment, especially after chemotherapy, is high in Chinese patients with lung cancer. The distribution of pathogenic bacteria showed the highest detection rate for Gram-negative bacteria. In the future, preventive interventions should be administered to reduce the incidence of pulmonary infections in lung cancer patients. Patients should be given more support therapy, improve the nutritional status of patients, enhance the immune capacity of patients, in order to reduce the chance of infection of pathogenic bacteria. For the prevention of fungal infection, attention should be paid to reducing the adverse effects of treatment measures on the suppression of immune function in patients.

## Data Availability

The data sets used and analyzed during the current study are available from the corresponding author on reasonable request.
